# Retinal Thickness Normative Data in Wild-Type Mice Using Customized Miniature SD-OCT

**DOI:** 10.1371/journal.pone.0067265

**Published:** 2013-06-27

**Authors:** Lee R. Ferguson, James M. Dominguez II, Sankarathi Balaiya, Sandeep Grover, Kakarla V. Chalam

**Affiliations:** 1 Department of Ophthalmology, University of Florida College of Medicine, Jacksonville, Florida, United States of America; 2 Department of Pharmacology and Therapeutics, University of Florida College of Medicine, Gainesville, Florida, United States of America; Institute Biomedical Research August Pi Sunyer (IDIBAPS) - Hospital Clinic of Barcelona, Spain

## Abstract

**Objective:**

To report normative data for retinal thickness in wild-type C57BL/6 mouse utilizing a miniature SD-OCT system.

**Methods:**

Thirty adult mice (range: 3–5 months) were anesthetized and secured into the Bioptigen Spectral Domain Ophthalmic Imaging System. Right eye SD-OCT images were standardized by centralizing the optic nerve head (ONH) prior to image acquisition. Global and quadrant total retinal thickness (TRT) values were measured from retinal nerve fiber layer to retinal pigment epithelial layer. Posterior segment analyses also included the outer retinal layer (ORL) and inner retinal layer (IRL). Further sublayer analyses of four layers from the ORL and three layers comprising the IRL were also performed.

**Results:**

The overall mean±SD global TRT in a C57BL/6 mouse model was 204.41±5.19 µm. Quadrant mean TRT values were 204.85±5.81 µm inferiorly, 204.97±6.71 µm nasally, 205.08±5.44 µm temporally, and 202.74±4.85 µm superiorly. Mean±SD thickness for ORL, and IRL were 126.37±10.01 µm, and 107.03±10.98 µm respectively. The mean±SD estimates for the four layers of the ORL were 18.23±2.73 µm, 26.04±4.21 µm, 63.8±6.23 µm, and 19.22±4.34 µm. Mean±SD values for the three IRL sublayers were 27.82±4.04 µm, 59.62±6.66 µm and 19.12±3.71 µm.

**Conclusion:**

This study established normative values for the total retinal thickness and sublayer thickness for the wild-type C57BL/6 mice. Moreover, it provides a standard of retinal morphology, in a commonly used animal model, for evaluating therapeutic interventions and retinal disease pathophysiology.

## Introduction

Spectral domain optical coherence tomography (SD-OCT) is an important imaging modality in clinical ophthalmology and vision science for characterizing morphology and understanding pathophysiological changes within the retina. The rapid, high resolution, non-invasive cross-sectional images produced by SD-OCT is an important tool for diagnosing and monitoring posterior segment pathology longitudinally [1], [2].

Mouse models have been instrumental for understanding pathophysiology in a variety of retinal diseases [3]–[21]. Historically, e*x vivo* histological sectioning is used to study retinal disease in mouse models. Inherent methodological limitations confound tissue integrity and curtail longitudinal evaluations within a single subject. With the advent of *in vivo* applications such as fundoscopy, confocal scanning laser ophthalmoscopy (cSLO), angiography, electroretinography (ERG), and optical coherence tomography (OCT), limitations associated with *ex vivo* histological preparations can now be circumvented. Although SD-OCT provides non-invasive histological – grade sections of the rodent posterior segment, image acquisition is technologically cumbersome as human devices are retrofitted for animal use.

Hand-held spectral domain ophthalmic imaging system (SDOIS) (Bioptigen, Inc., Durham, NC) represents an alternative non-invasive *in vivo* imaging device. It contains the animal imaging mount with rodent alignment stage (a unique platform with adjusters that stereotactically secures mice within a cassette while mounted to a rotational stage) and a hand-held 840 nm SD-OCT probe (HHP) capable of axial resolutions of 5 micrometers (µm) [22]–[25]. Bioptigen SD-OCT technology is increasingly implemented in rodents [26] for biometrical analysis as well as evaluation and management of retinal disorders. However, data on normative values of the retina for wild-type C57BL/6– a common mouse model for the study of retinal diseases – is not available. The overall goal of this study is to report normative SD-OCT data of the mouse retina with the Bioptigen SDOIS.

## Methods

### Ethics Statement

All experiments were performed in compliance with the University of Florida institutional animal care and use committee (IACUC) guidelines and/or adhered to the Association for Research in Vision and Ophthalmology Statement for the Use of Animals in Ophthalmic and Vision Research. This study was reviewed and approved by the University of Florida IACUC. All animals were managed in accordance with the University of Florida animal care services (ACS) rodent handling procedures. Animal wellbeing and welfare were monitored daily by the ACS staff and the study investigators. Ketamine/Xylazine anesthetic mixture was used to sedate animals during scanning in order to minimize distress. During the course of this study, no animal was sacrificed or showed signs of suffering.

### Animals

Thirty adult C57BL/6 mice (Jackson Laboratory, Bar Harbor, ME) between the ages of three to five months were utilized for this study. All mice were housed and maintained in the University of Florida ACS facility. The mice were adherent to a 12-hour light/dark cycle with open access to feed and water. Animal preparation and SD-OCT scanning were performed in an ACS-designated and approved procedural location.

### SD-OCT Imaging

**Figure 1:**
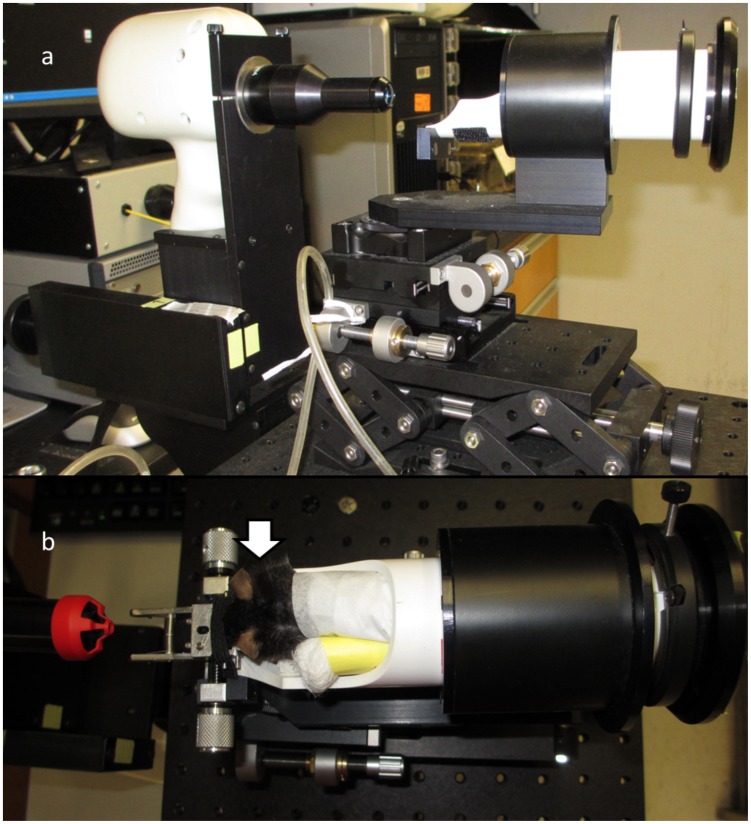
Imaging Apparatus. (a) The Bioptigen spectral-domain ophthalmic imaging system (SDOIS) with animal imaging mount - rodent alignment stage (AIM-RAS) components; (b) aerial view of mouse within cassette - white arrow points to right eye where all scans were obtained.

Mice were anesthetized with a mixture of Ketamine (Ketaject; 80 mg/kg; Webster Veterinary, Devens, MA) and Xylazine (Ana Sed; 10 mg/kg; Webster Veterinary; Devens, MA) delivered via intraperitoneal injection to ensure restriction of movement during optical scanning. Both pupils were dilated with topical Tropicamide (1%; Akorn Inc.; Lake Forest, IL). To prevent corneal desiccation during procedure, topical Systane Ultra® lubricant eye drop (Alcon, Fort Worth, TX) was applied bilaterally every minute. Following dilation and lubrication, the mice were positioned into the AIM-RAS setup (Figures 1a &1b), which permitted X, Y, and Z axis manipulation for proper alignment of mouse eye with HHP bore in order to properly center and visualize the ONH within the viewing panes for B-scan and en-face images.

In order to achieve ONH centering during imaging, the geometrical working distance between the subject eye and the HHP bore was acquired by an aiming tip and aiming target calibration method prior to mouse insertion into AIM-RAS apparatus. The working distance between the SD-OCT lens and the mouse eye was approximately 5 mm. In order to maintain central arrangement prior to SD-OCT image capture, the ONH was repositioned vertically by clockwise and counter clockwise animal cassette rotation and horizontally by swiveling the mouse cassette towards the nasal or temporal locations. Careful attention was paid to accurate focusing of the image at the time of manual calibrations to the RAS and mouse cassette. Once image acquisition commenced, the scans were performed in rapid succession without need for further manipulation of the mice. The imaging protocol entailed a 3 mm×3 mm perimeter square scan sequence producing a single en-face image of the retina through a 50-degree field of view from the mouse lens, following mydriasis. The en-face image comprised of 100 B-scan tomograms with each B-scan consisting of 1000 A-scans.

**Figure 2:**
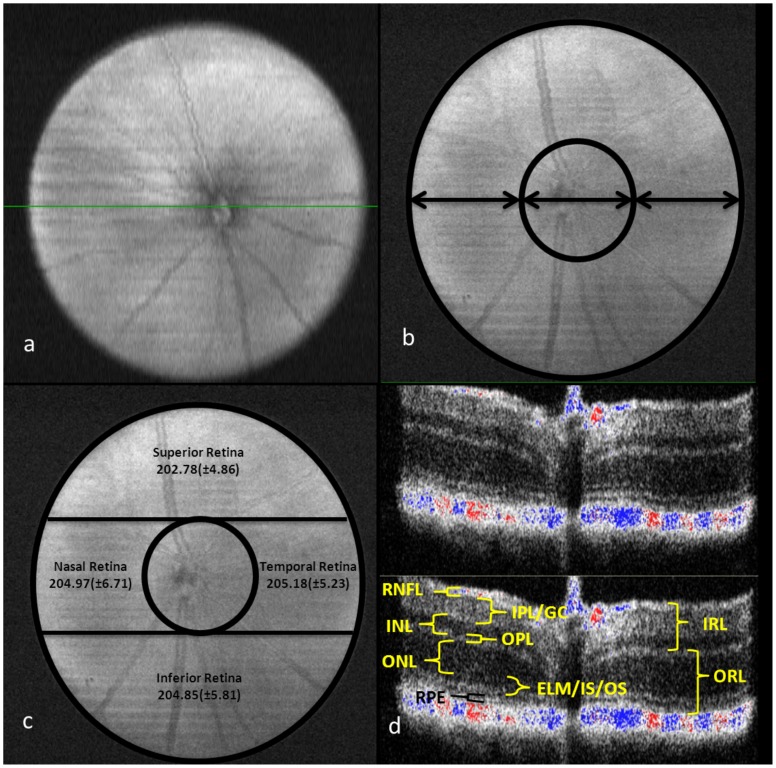
(a) C57BL/6 mouse en-face scan showing the central optic nerve head; (b) showing the 1 mm diameter (arrows) within and around central retinal subfield (CSF); (c) the retinal quadrants surrounding CSF with overall mean (±SD) total retinal thickness; (d) a B-scan image from equatorial slice of en-face scan showing the different sublayers that were measured using the manual calipers. [ORL, outer retinal layer; IRL, inner retinal layer; RPE, retinal pigment epithelium; ELM/IS/OS, external limiting membrane/inner segment of photoreceptors/outer segment of photoreceptors; ONL, outer nuclear layer; OPL, outer plexiform layer; INL, inner nuclear layer; IPL/GC, inner plexiform layer/ganglion cell; RNFL, retinal nerve fiber layer].

All scans were performed by one of two operators (LRF or JMD). B-scan slices were recorded only if posterior segment layers could clearly be resolved with set standardized image display options for both image brightness and contrast. SD-OCT scans were not recorded if retinal vasculature cross-sections were visualized within any of the measured segment layers. The en-face image was transected centrally along the ONH in order to provide a B-scan image of the retina layers at this location (Figure 2a). The ONH was used as the landmark with central subfield (CSF) scan diameter of approximately 1 mm (Figure 2b).

### Analysis of SD-OCT Imaging

The SD-OCT B-scan cross-sectional images were analyzed with the InVivoVue Clinic software incorporated with the SDOIS. The software included ‘manual calipers’ that could be used for measurements in microns (µm) of the various posterior segment sublayers. Retinal en-face scans were divided into inferior, nasal, temporal, and superior quadrants surrounding the retinal CSF containing the ONH (Figure 2c). The total retinal thickness (TRT) was defined as the width from the RNFL to the RPE layer, including both layers. Four measurements were made on the same scan and averaged. Within the center of each quadrant, three B-scans separated by approximately 150 µm were used for measurements. Hence, 12 points were used for each quadrant to average the retinal thickness measurements.

After determining the total retinal thickness in the retinal quadrants, measurements were made for the outer retinal layer and inner retinal layer. Further, retinal thickness sub-analyses were done to measure each individual layer of the outer and inner retina (Figure 2d) with manual calipers in the following order: outer retina – retinal pigment epithelium (RPE), external limiting membrane/inner segment of photoreceptors/outer segment of photoreceptors (ELM/IS/OS), outer nuclear layer (ONL), outer plexiform layer (OPL); inner retina – inner nuclear layer (INL), inner plexiform layer/ganglion cell (IPL/GC), and the retinal nerve fiber layer (RNFL). Although the recording of the scans were done on a single day for each mouse, the measurement of the TRT and the retinal thickness sub-layers were done at different times.

### Statistical Analysis

Mean and standard deviation (± SD) were calculated for the within-subject triplicate measurements from each layer of the posterior segment. The mean (± SD) between subject values for the posterior segment layers were then computed**.** Students’ T-test was used to calculate statistical significance and a p-value less than 0.05 was considered significant. We looked at the interobserver variability between two independent observers (LRF and WL) using two methods: first, by measuring TRT for all animals, and then the sublayer thickness values from ten randomly selected animals. The mean difference and 95% confidence interval (CI) for interobserver measurements were estimated. A two-way mixed effect intraclass correlation (ICC) assessment (SPSS.21) was used to measure interobserver agreement. Bland-Altman plots were constructed to assess the degree of interobserver agreement for TRT and sublayer measurements.

## Results

Retinal thickness measurements from a total of thirty adult mice eyes were analyzed. Globally, the mean±SD TRT value for all study mice was 204.41±5.19 µm (Table 1). The four outer quadrant retinal areas surrounding the CSF region had mean retinal thickness values of 204.85±5.81 µm inferiorly, 204.97±6.71 µm nasally, 205.08±5.44 µm temporally, and 202.74±4.85 superiorly. The overall mean thickness for the outer retina and inner retina were 126.37±10.01 µm, and 107.03±10.98 µm respectively. Thickness values for the retinal sublayers were: RPE = 18.23±2.73 µm; ELM/IS/OS = 26.04±4.21 µm; ONL = 62.8±6.23 µm; OPL = 19.22±4.34 µm; INL = 27.82±4.04 µm; IPL/GC = 59.62±6.66 µm; RNFL = 19.12±3.71 µm (Table 1).

**Table 1:** Mean Total Retinal Thickness and Sublayer Thickness±Standard Deviation (µm).

	n = 30
Total Retinal Thickness – Global	204.41±5.19
Total Retinal Thickness – Inferior	204.85±5.81
Total Retinal Thickness – Nasal	204.97±6.71
Total Retinal Thickness –Temporal	205.08±5.44
Total Retinal Thickness – Superior	202.74±4.85
Outer Retina Layer	126.37±10.01
Inner Retinal Layer	107.03±10.98
Retinal Pigment Epithelium	18.23±2.73
External Limiting Membrane/Inner Photoreceptor Segment/Outer Photoreceptor Segment	26.04±4.21
Outer Nuclear Layer	62.8±6.23
Outer Plexiform Layer	19.22±4.34
Inner Nuclear Layer	27.82±4.04
Inner Plexiform Layer/Ganglion Cell	59.62±6.66
Retinal Nerve Fiber Layer	19.12±3.71

Table 2 shows the mean global TRT and the retinal thickness in the four quadrants in each animal. Similarly, Table 3 depicts the mean retinal thickness of different sublayers in each animal.

**Table 2:** Mean Global Total Retinal Thickness (µm) and in Different Quadrants of Retina in Each Animal.

Animal	TRT-I	TRT-N	TRT-T	TRT-S	TRT-Global
1	204.17	203.67	205.00	198.83	202.92
2	206.33	200.67	206.67	200.00	203.42
3	203.33	203.67	203.00	202.17	203.04
4	202.67	208.33	201.00	199.83	202.96
5	203.17	208.00	200.67	202.33	203.54
6	205.00	205.67	204.33	200.83	203.96
7	199.00	197.33	200.00	195.50	197.96
8	199.67	204.33	204.67	196.67	201.33
9	203.33	201.33	207.33	204.50	204.13
10	198.67	195.00	200.00	196.50	197.54
11	199.83	190.00	201.33	203.00	198.54
12	199.00	194.67	193.00	193.00	194.92
13	203.00	208.00	207.00	205.50	205.88
14	209.50	205.33	202.67	202.17	204.92
15	230.33	225.00	225.00	216.33	224.17
16	208.00	213.00	213.67	209.00	210.92
17	201.33	205.33	201.67	206.17	203.63
18	209.67	207.00	203.33	204.17	206.04
19	200.00	203.33	205.33	200.50	202.29
20	202.50	196.00	205.00	200.17	200.92
21	203.83	204.00	204.67	199.67	203.04
22	206.17	203.67	205.33	204.50	204.92
23	203.17	207.67	206.00	204.00	205.21
24	204.50	207.33	203.00	206.17	205.25
25	202.83	202.67	202.00	202.33	202.46
26	207.00	210.00	210.00	202.00	207.25
27	206.50	214.00	208.00	206.83	208.83
28	209.83	205.00	204.33	203.33	205.63
29	204.50	205.67	206.00	202.67	204.71
30	208.67	213.33	212.33	213.67	212.00

Total retinal thickness (TRT) consists of all retinal layers including retinal pigment epithelium (RPE). TRT-I, total retinal thickness-inferior quadrant; TRT-N, total retinal thickness-nasal quadrant; TRT-T, total retinal thickness-temporal quadrant; TRT-S, total retinal thickness-superior quadrant.

**Table 3:** Mean Retinal Thickness (µm) in Different Sublayers In Each Animal.

Animal	RPE	ELM/IS/OS	ONL	OPL	INL	IPL/GC	RFNL	ORL	IRL
1	15.33	20.67	75.33	14.00	27.67	51.00	23.33	124.67	103.33
2	17.00	26.33	54.00	19.33	27.33	62.00	16.00	116.33	104.67
3	16.33	26.00	62.00	17.00	32.00	48.67	23.33	121.33	104.67
4	23.00	34.67	59.33	26.67	28.00	57.00	15.33	143.67	102.00
5	12.67	25.00	57.00	18.67	28.33	51.33	21.33	115.00	101.00
6	19.67	26.67	59.00	19.33	26.00	56.00	19.00	125.00	103.67
7	19.67	28.00	58.00	24.33	28.33	62.00	20.67	129.00	111.00
8	21.33	20.67	63.67	18.67	26.67	68.33	24.00	123.67	120.00
9	22.33	35.67	71.00	21.00	26.00	61.67	19.67	148.67	107.67
10	21.33	27.67	65.67	16.67	24.67	60.67	21.00	130.33	105.33
11	21.67	27.67	63.67	18.33	23.00	61.33	20.00	131.33	106.00
12	20.33	20.33	56.67	27.33	22.33	56.00	11.67	123.67	90.33
13	14.67	25.33	64.67	13.67	25.67	52.67	14.00	120.00	92.33
14	19.67	24.33	61.67	13.00	26.33	55.00	11.33	118.67	93.33
15	18.33	27.33	64.00	21.00	33.00	61.67	18.33	130.67	116.67
16	12.00	28.33	61.33	17.67	20.33	52.00	21.00	120.00	94.33
17	19.00	28.33	85.33	28.33	32.67	76.33	24.67	160.00	133.67
18	16.00	20.33	65.67	18.33	36.33	71.67	21.67	121.00	129.33
19	18.00	31.33	61.67	18.33	29.67	62.33	20.67	130.33	113.67
20	15.33	26.33	64.33	17.00	26.33	56.33	18.33	125.33	101.67
21	17.33	26.33	58.67	19.67	28.33	57.33	15.00	122.67	101.33
22	19.00	29.00	64.00	20.00	30.00	60.67	19.00	132.00	108.67
23	18.33	20.33	67.33	16.33	17.33	55.67	20.33	121.00	92.67
24	18.00	29.00	63.67	14.67	27.00	56.67	17.33	124.33	102.00
25	16.33	29.67	57.00	17.33	26.67	63.67	18.33	118.33	109.00
26	18.33	23.67	62.67	17.33	32.67	62.33	20.00	124.67	116.00
27	17.33	23.67	63.00	31.00	32.33	75.00	27.33	134.33	131.67
28	22.33	16.67	61.33	18.33	27.00	55.33	19.33	118.33	101.67
29	17.33	24.00	55.33	15.67	29.67	56.67	17.33	113.33	104.67
30	19.00	28.00	57.00	17.67	33.00	61.33	14.33	123.33	108.67

RPE, retinal pigment epithelium; ELM/IS/OS, external limiting membrane/inner segment of photoreceptors/outer segment of photoreceptors; ONL, outer nuclear layer; OPL, outer plexiform layer; INL, inner nuclear layer; IPL/GC, inner plexiform layer/ganglion cell; RNFL, retinal nerve fiber layer; ORL, outer retinal layer; IRL, inner retinal layer.

**Figure 3:**
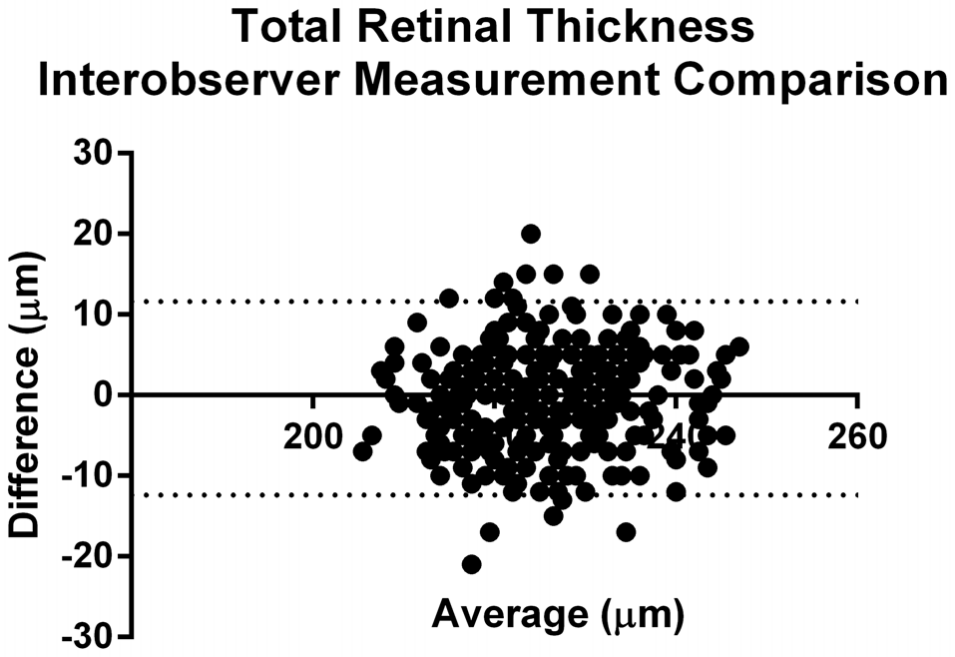
Bland-Altman plot showing representative inter-observer difference for total retinal thickness (TRT) measurements in the nasal quadrant of all animals (n = 30). Each black dot in the figure corresponds to a specific TRT measurement point within the B-scan – twelve points were measured in each quadrant. Since the graph depicts TRT values in the nasal quadrant for all 30 animals, in total, 360 TRT measurements are represented in this graph.

**Figure 4:**
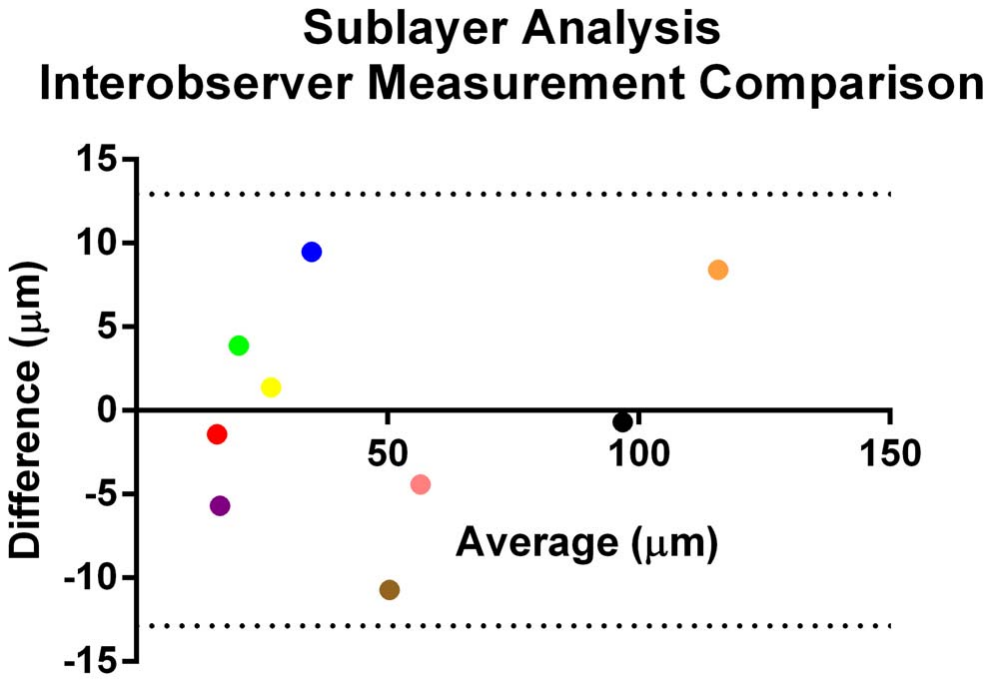
Bland-Altman plot showing inter-observer difference for mean retinal sublayer thickness measurements in 10 randomly selected animals. Each dot in the figure corresponds to the mean thickness from 10 animals and the color code represents 9 different sublayers: red, retinal pigment epithelium; blue, external limiting membrane/inner segment of photoreceptors/outer segment of photoreceptors; pink, outer nuclear layer; purple, outer plexiform layer; yellow, inner nuclear layer; brown, inner plexiform layer/ganglion cell; green, retinal nerve fiber layer; orange, outer retinal layer; black, inner retinal layer.

In order to look for interobserver variability, as mentioned in Methods, we had two independent observers go back and measure the TRT in all animals. There was excellent interobserver agreement, as depicted in Figure 3, with an ICC of 0.88 (p<0.001, 95% CI = 0.76–0.94). Also, as seen in Figure 3, the majority of variability between observer TRT measurements was less than 10 µm. We also measured interobserver variability in sublayer retinal thicknesses, as shown in Table 4. Again, there was excellent interobserver agreement with an ICC value of 0.99 (p<0.001, 95% CI = 0.99–1). Here too, the observers showed remarkable consistency with layer measurements (Figure 4).

**Table 4:** Interobserver Correlation In Measurement of SD-OCT Mean Retinal Thickness (µm).

Layers	Observer 1	Observer 2	Mean Diff (Absolute Value)	95% CI
Outer Retina Layer	120± (6.25)	111.6± (2.63)	8.4	113–119
Inner Retinal Layer	96.4± (4.38)	97.1± (5.51)	0.7	95–99
Retinal Pigment Epithelium	15.3± (2.41)	16.7± (4.03)	1.4	15–17
External Limiting Membrane/Inner PhotoreceptorSegment/Outer Photoreceptor Segment	39.6± (3.78)	30.1± (6.57)	9.5	32–38
Outer Nuclear Layer	54.3± (8.07)	58.7± (6.33)	4.4	53–60
Outer Plexiform Layer	13.8± (1.62)	19.5± (3.60)	5.7	15–18
Inner Nuclear Layer	27.5± (4.01)	26.1± (3.41)	1.4	25–28
Inner Plexiform Layer/Ganglion Cell	45± (6.43)	55.7± (7.09)	10.7	47–54
Retinal Nerve Fiber Layer	22.3± (8.73)	18.4± (2.72)	3.9	17–23

## Discussion

SD-OCT technology has expanded the boundaries of vision science as it relates to non-invasive imaging of human retina. In human studies, SD-OCT normative values have been established for macular thickness [27], [28] and its sublayers [29]. This has served to further enhance the understanding of retinal architecture and structural deficits. With the use of SD-OCT, multiple, reproducible, high-resolution scans, accuracy in identifying defects at the micron level has improved. Moreover, serial imaging over time has also helped in studying the course of diseases and their prognoses.

Similarly, although the advent of this technology has heralded a revolution for animal study in vision science, normative database from a ubiquitous small animal species used to model the human eye has not been established. Various groups have assessed mouse TRT and retinal sublayers by utilizing different imaging setups. Some have measured these layers with retrofitted human SD-OCT devices coupled with an external lens to neutralize mouse corneal optical power [22], [23]. Other groups have incorporated custom-designed SD-OCT instruments that maintain resolution capabilities, while eliminating the need for external lens, when imaging rodent retinas [23], [25], [26].

In this present study, we determined the mean TRT measurement for the four retinal quadrants surrounding the ONH. Additionally, our findings included the mean thickness values for the individual layers that comprise the posterior segment in the C57BL/6 mouse strain, while utilizing a miniaturized SD-OCT with stereotactic multi-axis rotational rodent containment device (*i.e.* Bioptigen SDOIS).

In terms of TRT for the four retinal quadrants surrounding the ONH, our data suggest that there is uniformity in measurement value. Despite not reaching a significant difference, the superior retina region (202.74±4.85 µm) showed a trend of being the thinnest region as compared to inferior (204.85±5.81 µm), nasal (204.97±6.71 µm), and temporal (205.08±5.44 µm) areas. We estimated that the TRT for the C57BL/6 mouse – as extrapolated from averages of quadrant measurements– to be 204.41±5.19 µm. The results from our study are consistent with those obtained by previous groups [25], [30]. There have been other investigators who have shown mean TRT values ranging from 200–250 µm [22], [30], [31]. However, the measurement values obtained by Gabriele *et al*. (2010) [26] overestimates all prior mentioned reports. In addition to assessing global TRT, their report was the first to examine quadrant TRT in the C57BL/6 mouse. They employed a similar SD-OCT device, as in our study (Bioptigen Inc., Durham, NC), to generate en-face volumetric images, centered at the ONH. In the Gabriele *et al.* study, automated segmentation software was used to measure B-scan TRT spanning from the inner limiting membrane (ILM) to the RPE. Global and quadrant TRT outcome measurements were estimated by applying a linear mixed-effects statistical model. From their analyses, the authors reported that the mean global TRT was 298.21 µm [26]. Moreover, they showed the superior retina (310.38 µm) as being the thickest quadrant in comparison to the inferior (291.55 µm), nasal (296.52 µm) and the temporal (294.37 µm) zones. Higher TRT values obtained in that study could potentially be attributed to the region of selection indicated by automated segmentation. Although Gabriele *et al.* selected boundary points that supposedly corresponded to the ILM and RPE, it is possible that the choroid was factored in when measuring the retinal distance from the ILM to the RPE (Figure 3, Gabriele et al., 2010). This inclusion could have increased the global and quadrant TRT values in their study. Another probable explanation for their overestimated TRT values could be based on the level of resolution obtained with their B-scan images. Their scanning protocol required only 250 averaged A-scans (each an average of 4 individual A-scans)×250 frames, whereas our scanning protocol included 1000 A-scans per B-scan with 100 accumulated B-scans with a higher resolution.

In addition to TRT measurements, we also performed sub-analyses of the thickness values for different layers of the retina. Broadly, we first analyzed the thickness of the outer retina (126.37±10.01 µm) and inner retina (107.03±10.98 µm). Further, we also analyzed each of the retinal layers constituting the C57BL/6 retina. Although retinal sublayer measurements were also performed in a study by Fischer et al. (2009) [23], they investigated five retinal sublayers as compared to the seven evaluated in our study. Their study showed slightly increased retinal thickness as compared to our findings. This difference could possibly be accounted for by the dissimilarity in resolution – they used a third generation, commercially available human SD-OCT apparatus versus the SDOIS Bioptigen system used in our study. Another factor that could possibly account for the difference is the absence of sharp demarcation of each layer and hence, the inconsistency in defining different layer borders and their measurements. This inconsistency could be a possible explanation for the difference of about 30 µm in the mean global TRT (∼204 µm) and the sum of the mean outer retinal thickness (∼126 µm) and the mean inner retinal thickness (∼107 µm) in our study. These two measurements were done on two different days and a slightly different demarcation line may have been used for the actual measurements. Further improvement of resolution of OCT instrumentation and/or inclusion of automated calipers (unlike our study where manual calipers were used) may help better define the dimensions of various sublayers of the retina in the future. Despite this slight limitation, we were able to estimate standard deviations within a 10% margin of the mean for each TRT quadrant; this reflects a narrow range of variability. In addition, the two independent raters showed excellent agreement for measurements of TRT and the various sublayers.

In conclusion, this current study describes the normative retinal values for the C57BL/6 mouse while utilizing the Bioptigen SDOIS device. It provides normative values for the total retinal thickness as well as the individual sublayers of the retina. This will serve as a standard for disease models in this mouse strain to better understand the causes of diseases, their pathophysiological mechanisms, and any treatment options that maybe available.
